# Drug repurposing in Rett and Rett-like syndromes: a promising yet underrated opportunity?

**DOI:** 10.3389/fmed.2024.1425038

**Published:** 2024-07-29

**Authors:** Claudia Fuchs, Peter A. C. ‘t Hoen, Annelieke R. Müller, Friederike Ehrhart, Clara D. M. Van Karnebeek

**Affiliations:** ^1^EURORDIS – RARE Disease Europe, Paris, France; ^2^Center for Molecular and Biomolecular Informatics, Radboud Institute for Molecular Life Sciences, Radboud University Medical Center, Nijmegen, Netherlands; ^3^Department of Pediatrics, Emma Children’s Hospital, Amsterdam Gastroenterology Endocrinology Metabolism, Amsterdam UMC Location University of Amsterdam, Amsterdam, Netherlands; ^4^Amsterdam UMC, Emma Center for Personalized Medicine, Amsterdam, Netherlands; ^5^Department of Human Genetics, Amsterdam Reproduction and Development, Amsterdam UMC Location University of Amsterdam, Amsterdam, Netherlands; ^6^Department of Bioinformatics – BiGCaT, Research Institute of Nutrition and Translational Research in Metabolism (NUTRIM), Maastricht University, Maastricht, Netherlands

**Keywords:** Rett syndrome, CDKL5 deficiency disorder, FOXG1-syndrome, shared molecular pathways, common drug targets, drug repurposing

## Abstract

Rett syndrome (RTT) and Rett-like syndromes [i.e., CDKL5 deficiency disorder (CDD) and FOXG1-syndrome] represent rare yet profoundly impactful neurodevelopmental disorders (NDDs). The severity and complexity of symptoms associated with these disorders, including cognitive impairment, motor dysfunction, seizures and other neurological features significantly affect the quality of life of patients and families. Despite ongoing research efforts to identify potential therapeutic targets and develop novel treatments, current therapeutic options remain limited. Here the potential of drug repurposing (DR) as a promising avenue for addressing the unmet medical needs of individuals with RTT and related disorders is explored. Leveraging existing drugs for new therapeutic purposes, DR presents an attractive strategy, particularly suited for neurological disorders given the complexities of the central nervous system (CNS) and the challenges in blood-brain barrier penetration. The current landscape of DR efforts in these syndromes is thoroughly examined, with partiuclar focus on shared molecular pathways and potential common drug targets across these conditions.

## 1 Introduction

Rett syndrome (RTT, #312750) and Rett-like syndromes, e.g., CDKL5 deficiency disorder (CDD, #300672) and FOXG1-syndrome (or FOXG1-related encephalopathy, #613454) are rare monogenic neurodevelopmental disorders (NDDs). The relative recent recognition of their distinct clinical entities (1, 2) has deepened our understanding of their underlying pathogenic mechanisms and clinical characteristics (Table 1). Although each disorder exhibits unique clinical features, they share common core symptoms and neurological traits (Table 1), suggesting that these disorders share critical molecular etiology.

**TABLE 1:** Genetic, molecular and symptomatic aspects of Rett syndrome (RTT) and Rett-like syndromes, i.e., CDKL5 deficiency disorder (CDD) and FOXG1-syndrome.

	Genetics	Function and molecular effects	Clinical features
Rett syndrome (RTT)	• Caused by mutations in the X-linked methyl CpG-binding protein 2 gene *(MECP2*; Xq28) – In 90 - 95% of cases RTT is caused by loss-of-function mutations in MECP2 (100) – Despite hundreds of different mutations in MECP2 have been identified, eight hotspot mutations account for more than 60% of all cases (101) – MECP2 mutations arise predominantly in the paternal germ line (102)	• MeCP2 is a nuclear protein highly expressed in the brain • MeCP2 is a multifunctional “hub” involved in numerous pathways that support brain function, acting as transcriptional repressor or activator (depending on the context), facilitating chromosome looping or compaction, regulating miRNA processing or splicing (103)	• Incidence: 1/10.000 live births (second most common cause of intellectual disability in females). RTT mainly affects females. • RTT patients exhibit apparently typical early postnatal development followed by rapid developmental regression/ stagnation (commonly the first signs appear of 6–18 months of age) • The hallmark symptoms of RTT include: – Verbal and nonverbal communication deficits > partial or complete loss of purposeful hand movements, stereotypic hand movements, loss of speech – Loss of motor skills – Gait abnormalities – Severe intellectual disability, (seizures) – Breathing abnormalities, sleep problems, gastrointestinal problems, …
CDKL5 deficiency disorder (CDD)	• Caused by mutations in the X-linked cyclin-dependent kinase-like 5 gene (*CDKL5*, Xp22.13) – Most pathogenic missense mutations cluster in the highly conserved N-terminal catalytic domain (contains an ATP-binding side, an activation side and a TEY motif activation loop), suggesting that the enzymatic activity of CDKL5 is essential for normal brain development/ function.	• CDKL5 is a serine/threonine kinase. CDKL5 shows homologies to serine/threonine kinase and was initially named as STK9 (serine/threonine kinase 9). • CDKL5 is highly expressed in the brain; predominantly in neuronal nuclei and dendrites, with peak expression in early postnatal life (12), when symptoms typically begin. • CDKL5 has been implicated in several essential neuronal functions through interaction or association with other proteins, or by direct phosphorylation/modification of target proteins [reviewed in (11, 12)].	• Incidence: 1/40.000−60.000 live births. Female-to-male ratio 4:1 (23). • Initially known as early seizure or Hanefeld variant of RTT, now considered an independent clinical entity (1). • CDD clinical symptoms overlap in part with those of RTT but distinguish for one prominent and unique feature: epileptic seizures usually begin within the first 3 months of life (median age of refractory epilepsy) onset is 6 weeks with 90% onset by 3 months, (1, 22, 23) • Clinical features of CDD include (23): – Severe global developmental delays and intellectual disability (no regression period) – Stereotypic hand movements, repetitive leg crossing – Loss of speech – Loss of motor skills and movement disorders – Cortical visual impairment – Breathing abnormalities, sleep problems, gastrointestinal problems, …
FOXG1-syndrome	• Caused by mutations in the forkhead box G1 gene (*FOXG1*; 14q12) (FOXG1-syndrome is generally caused by mutations within the FOXG1 gene itself, while other results from a deletion or duplications of genetic material from a region of the long (q) arm of chromosome 14 (104).	• FOXG1 is a transcription factor that serves as a master regulator for brain development. FOXG1 interacts with multiple signaling pathways and is essential for the proliferation of the progenitor cells of the cerebral cortex (2, 13).	• Incidence: 1/30.000 live births. • Initially known as congenital variant of RTT. • Over the past years increasing reports on individuals harboring FOXG1 mutations have allowed further expansion and delineation of the clinical phenotypes of FOXG1 mutations, thus progressing parting from RTT. Nevertheless, a combination of developmental and anatomical features distinguishes FOXG1-syndrome from the typical RTT. Individuals with FOXG1-syndrome show a more severe clinical phenotype compared to RTT. • Core clinical features of FOXG1syndrome include (2): – Microcephaly – Severe developmental delay and cognitive disability – Absence or minimal language development – Complete lack of “eye gaizing/pointing” – Early-onset dyskinesia and hyperkinetic movements – Stereotypies – Seizures – Cerebral malformations

The table outlines the genetic cause of each disorder and provides a brief overview of the molecular pathways involved and the hallmark symptoms typically observed in affected individuals. Although each syndrome/disorder exhibits unique clinical features, they share common core symptoms and neurological traits, suggesting that these disorders share a critical molecular etiology. This commonality underscores the interconnectedness of these conditions at the molecular level and highlights the potential for shared pathways and targets for therapeutic interventions.

Identifying shared pathways holds significant implications for targeted therapies development and drug repurposing (DR). DR, which involves using existing drugs for new therapeutic purposes, represents a promising approach in the treatment across multiple diseases especially for neurological disorders (3, 4). The complex structure of the central nervous system (CNS), coupled with the challenge of penetrating the blood-brain barrier, poses significant hurdles in the development of new drugs for neuropathological conditions, making DR of particular interest for these disorders. Notable successes of DR in NDDs include e.g., repurposing of fenfluramine in Dravet syndrome (5) or bumetanide (6) and pregnenolone (7) for autism spectrum disorders. These studies validate DR as a valid treatment approach for multiple neuropathological conditions.

We here discuss the current state of art of DR efforts in RTT, CDD and FOXG1-syndrome, with particular emphasis on the shared molecular pathways and the identification of common drug targets across the three conditions. For a more detailed overview on the molecular and circuit mechanisms underlying each syndrome, please refer to (8–10) for RTT, (11, 12) for CDD and (2, 13) for FOXG1-syndrome (2, 13).

## 2 Molecular mechanisms underlying RTT, CDD, and FOXG1-syndrome

**FIGURE 1:**
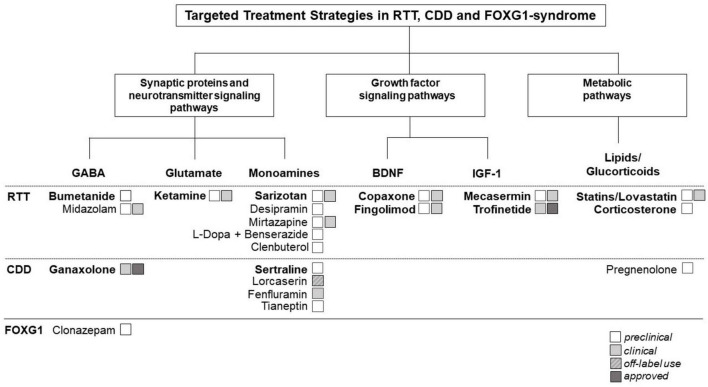
Therapeutic targets and potential repurposing strategies currently being explored in Rett syndrome (RTT) and Rett-like syndromes [i.e., CDKL5 deficiency disorder (CDD)] and FOXG1-syndrome. Shared molecular pathways across these disorders can be categorized in three main categories: (1). Synaptic proteins and neurotransmitter signaling pathways. (2). Growth factor signaling pathways. (3). Metabolic pathways. The figure illustrates the interconnectedness of molecular pathways across these disorders, highlighting common themes and potential targets for therapeutic intervention. Understanding these shared pathways may guide the development of repurposing strategies aimed at addressing the underlying molecular mechanisms of these neurodevelopmental disorders (NDDs). The figure highlights also the current repurposed drugs approved or being investigated for the treatment of RTT, CDD, and/or FOXG-1 syndrome. In bold, repurposing candidates described in detail in the main text of the manuscript.

As shown in Figure 1, pathways across the three diseases can be categorized in three main categories.

### 2.1 Synaptic proteins and neurotransmitter signaling pathways

Synaptic proteins and neurotransmitter signaling pathways are crucial for correcting brain function. Disruptions in these components are linked to alterations in excitatory/inhibitory (E/I) balance, synaptic transmission, and neuronal network activity: common features in many NDDs (14, 15). Neuronal hyperexcitability is a common causative feature contributing to the high prevalence of seizures observed in all three disorders (Table 1). It is intricately linked to the “GABA switch” phenomenon (16): during critical stages of brain development, GABA initially exerts excitatory effects due to high intracellular chloride concentrations. As development progresses, the expression and activity of involved chloride co-transporters shifts, leading to a corresponding change in the polarity of GABAergic signaling from excitatory to inhibitory. Disruptions in this transition, such as delayed maturation or altered expression of chloride co-transporters, can prolong the excitatory phase of GABAergic signaling, resulting in neuronal hyperexcitability. This phenomenon has been extensively studied in NDDs, including RTT (17–20). Multiple studies have investigated the effects of NKCC1 inhibition by the FDA-approved diuretic bumetanide as therapeutic option for multiple NDDs (21) (see section 3.1 - Bumenatide).

Epilepsy is prevalent in RTT and RTT-like disorders (Table 1), particularly in CDD, where seizures commonly manifest within the first 3 months of life (22, 23). Ganaxolone, a synthetic analogue of the neurosteroid allopregnanolone, which is a positive allosteric modulator of the GABA_A_ receptor, has recently been approved for use in CDD, showing promise in managing seizures (see section 3.2 - Ganaxolone).

Dysfunctions in GABAergic signaling pathways contribute to various other RTT-related symptoms, including breathing abnormalities. Respiratory abnormalities in RTT mouse models can be significantly improved by manipulating diverse neurotransmitter systems (24, 25). Benzodiazepine like midazolam, generally used for anesthesia and procedural sedation, alleviate breathing defects in MeCP2-null mice (26) and has also been used for the acute management of prolonged seizures in RTT (27). Interestingly, Chen et al. showed that treatment with clonazepam, a long-acting tranquilizer of the benzodiazepine class, alleviates cognitive defects, limited social interactions, and depression-like behaviors in FOXG1 mutant mice (28).

In addition, changes in excitatory glutamatergic pathways and monoamine neurotransmitters, such as serotonin, dopamine and noradrenaline play a significant role in RTT and RTT-related disorders (26, 27) and several drugs targeting these changes have been tested. Of particular interest is ketamine, a NMDAR antagonist used as dissociative anesthetic and currently repurposed for the treatment of RTT (see section 3.3 - Ketamine).

Among the different monoamine neurotransmitters implicated especially serotonin has gained great attention in RTT and CDD. Different groups have shown a complex dysregulation of the serotoninergic system in mice and individuals with RTT and CDD [RTT: (29–31), CDD: (32, 33)]. Serotoninergic dysregulation in the brainstem contributes to breathing abnormalities and sarizotan, a 5-HT1A and D2-Like Receptor agonist, has been tested on respiratory dysfunction in RTT (34) (see section 3.4 - Sarizotan). A preclinical repurposing study showed that treatment with sertraline, a selective serotonin reuptake inhibitor (SSRI) and commonly used antidepressant improves behavioral abnormalities in CDD mice (33) (see section 3.5 - Sertraline). Many other antidepressants/drugs targeting the serotonergic pathway, including desipramine (35, 36) and tianeptine (37) have shown promising results in RTT and CDD models [reviewed in (35, 36)]. Interestingly, the antidepressant mirtazapine (acts as a potent antagonist of 5-HT2A and 5-HT3 receptors) was shown to ameliorate motor and social behaviors in RTT mice and patients (38, 39).

Different other drugs targeting monoamine neurotransmitter defects in RTT mice were tested. Szczesna and colleagues used the combination of L-Dopa with the dopa-decarboxylase inhibitor (Ddci) benserazide, a combination frequently used in Parkinsonism and related disorders, to stimulate dopaminergic deficiency in RTT mice (37). Mello and colleagues showed that treatment with the adrenergic receptor agonist clenbuterol, primarily used as a bronchodilator to treat conditions like asthma and chronic obstructive pulmonary disease (COPD), increased survival and improved behavioral deficits in RTT mice (40).

### 2.2 Growth factor signaling pathways

Dysregulations in growth factor signaling pathways has been widely implicated in the pathogenesis of RTT and RTT-like disorders. The role of brain-derived neurotrophic factor (BDNF) in promoting neuronal and synaptic development and function [reviewed in (38, 39)], as well as its implication in RTT [reviewed in (41)] and CDD (42) have been widely described. The involvement of BDNF dysregulation in the pathogenesis of FOXG1-syndrome remains to be elucidated (43). Several drugs targeting BDNF dysregulation have been repurposed in addition to different SSRIs and tianeptine, known to also enhance BDNF expression: BDNF boosters such as glatiramer acetate (copaxone) and fingolimod were recently investigated in RTT (see section 3.6 - BDNF-boosters).

Similarly, insulin-like growth factor 1 (IGF-1) is essential for neuronal maturation, survival, and plasticity [reviewed in (44)]. Alterations in IGF signaling pathways have been investigated in both mice and humans with RTT (45) and advanced the development of potential therapeutic strategies targeting IGF signaling in RTT. Interestingly, several studies in RTT mice have shown, that recombinant human IGF-1 (rhIGF-1) rescues RTT-related phenotypes in mice (46, 47). These experimental findings have led to a phase I and II clinical trial with rhIGF-1 (mecaserim) and two phase II trials with the IGF1 peptide analogue trofinetide in RTT patients (see section 3.7 - IGF-1).

### 2.3 Metabolic pathways

Several studies have shown that perturbations in metabolic pathways, influence neurological and non-neurological features in RTT [reviewed in (48–50)]. Alterations in the blood metabolite levels associated with RTT, support the concept that RTT and RTT-related disorders are systemic diseases affecting neurodevelopment.

Abnormalities in lipid metabolism and alterations in lipid profiles, including changes in cholesterol levels have been found in mice and individuals with RTT [reviewed in (51)]. Some studies have suggested that statins, a class of medications primarily used to lower cholesterol levels in the blood may have neuroprotective effects and could potentially offer benefits in RTT and RTT-like syndromes (see section 3.8 - Statins/Lovastin). Disruptions in metabolic pathways are very complex in RTT and RTT-like disorders and offer a broad array of potential therapeutic targets beyond statins. Several studies have investigated abnormalities in brain glucose metabolism in individuals with RTT (52), and investigated the possibility to pharmacologically intervene using for example glucocorticoid (see section 3.9 - Glucorticoids/Corticosterone).

## 3 Examples of repurposed drugs in RTT and RTT-related disorders

In this section, we will explore specific examples of repurposed drugs used in RTT and RTT-related disorders, drawing from our experience and understanding of common pathways (Figure 1). Rather than conducting a comprehensive review, we will highlight notable instances of DR within this context.

### 3.1 Bumetanide

The FDA-approved diuretic bumetanide, acting as an NKCC1 chloride importer antagonist, has garnered great interest for its potential in multiple NDDs (21, 53, 54). Lozovaya et al. have recently demonstrated that in a RTT mouse model the GABA shift is abolished at birth and that this alteration persistent in juvenile offspring can be alleviated by maternal administration of bumetanide (55), suggesting that repurposing of bumetanide might be promising in RTT. However, its use across multiple neurological disorders faces several limitations: bumetanide has low brain penetration and is associated with several collateral issues due to excessive diuresis caused by NKCC2 inhibition in the kidney (21). Ongoing drug discovery efforts seek to develop novel NKCC1 inhibitors and modulators with fewer adverse effects (21) and evaluate their potential across multiple NDDs, including RTT and RTT-related disorders.

### 3.2 Ganaxolone

Ganaxolone (3-hydroxy-3β-methyl-5δ-pregnane-20-one) belongs to a novel group of neuroactive steroids called epalons that act as positive allosteric modulators of GABA_A_ receptors to enhance GABAergic inhibition (56). FDA approved since 1983, ganaxolone has been investigated in various indications, including epilepsy and psychiatric disorders (57–61). A recent phase III trial (NCT03572933) has shown that ganaxolone reduces the frequency of CDD-associated seizures and is generally well tolerated (62, 63). Ganaxolone has been approved (US: March 2022, Europe: July 2023) for the treatment of seizures in CDD patients aged 2 years and older.

### 3.3 Ketamine

Channel-blocking NMDAR antagonists such as ketamine have shown significant promise in addressing the complex E/I imbalance associated with RTT and RTT-like disorders. Several preclinical studies have shown that ketamine improves cognitive function and behavior in animal models of RTT (64). Ketamine, a dissociative anesthetic initially used for induction and maintenance of anesthesia and repurposed for depression and related disorders (65) has shown promising results in RTT patients. A recently completed phase II clinical trials (NCT03633058) to assess the safety, tolerability and efficacy of low-dose oral ketamine in RTT patients has shown improvements across a broad range of symptoms (unpublished data). Ketamine has currently (in February 2023) been granted Orphan Drug Designation by the FDA and a phase III clinical trial is planned.

### 3.4 Sarizotan

Although sarizotan, a phase III 5-HT1A receptor agonist used for the treatment of dyskinesias in Parkinson’s disease (PD) (66, 67) has shown beneficial effects on respiratory dysfunction in different RTT models (34), a phase III clinical trial conducted in 2020 (NCT02790034) has not confirmed these results in a clinical setting. Low and high dose daily oral administration of sarizotan was ineffective in meeting its primary goal of reducing the percentage of apneas in children and adults with RTT-related breathing abnormalities. A new chemical entity acting as selective 5-HT1A agonist (NLX-101), which has shown antidepressant and cognitive enhancing properties, is currently under development (68, 69).

### 3.5 Sertraline

Sertraline, a widely used antidepressant to treat panic, generalized and social anxiety and obsessive compulsive disorders has been tested in a preclinical setting for CDD (70). The study has shown that treatment with sertraline improved several behavioral abnormalities in CDD mice (70). As sertraline is currently not approved for the use in children, it is widely described “off-label” for certain mental health conditions (71, 72). Different studies have evaluated the long-term impact of treatment with sertraline on cognitive, emotional and physical development in pediatric subjects (73–75) and the drug seems quite well tolerated. Interestingly, the sertraline study also included preliminary results on the off-label use of lorcaserin in CDD patients (33). Locaserin, like fenfluramine, acts as a selective 5-HT2C receptor agonist and was initially developed and approved as appetite suppressor. Although the use of both medications was discontinued in the treatment of severe obesity, due to safety and toxicity concerns, lorcaserin and fenfluramine are currently repurposed for the treatment of seizures associated with Dravet syndrome and Lennox-Gastaut syndrome [reviewed in (5, 76)]. A phase II open label trial to investigate the effects of repurposing of fenfluramine to control seizures in CDD patients is currently ongoing (NCT03861871) and preliminary results were published (77).

### 3.6 BDNF-boosters

#### 3.6.1 Glatiramer acetate

Glatimarer acetate, sold under the name copaxone is an immunomodulator medication to treat multiple sclerosis (MS). As glatiramer acid stimulates the secretion of BDNF in the brain and the levels of BDNF expression seem to be directly correlated with the severity of RTT related symptoms (78), a small phase II clinical trial (NCT02153723) was run in 2014. Although the trial showed improvements in gait analyses, respiratory dysfunction, electroencephalographic findings, and quality of life, there were severe safety concerns (79, 80).

#### 3.6.2 Fingolimod

Like copaxone, fingolimod is an immunomodulating medication used for the treatment of MS. Interestingly this sphingosine-1-phosphate analog, has been shown to upregulate BDNF mRNA levels and increase BDNF protein release (81) and to increase cognitive impairment in Huntington’s disease and Alzheimer’s diseases (82, 83). A recent phase II clinical trial to assess safety and efficacy of fingolimod in children with RTT (NCT02061137) has shown that treatment with fingolimod was safe but had no significative effects on RTT-associated symptoms (84).

### 3.7 IGF-1

#### 3.7.1 Mecasermin (recombinant human IGF-1, rhIGF-1)

Mecasermin is FDA-approved for the long-term treatment of growth failure in children with severe primary IGF-1 deficiency (85). A phase I and II clinical (NCT01777542) study assessed safety and efficacy of mecasermin treatment in RTT patients. Both studies (86, 87) reported that a significant proportion of RTT patients exhibited limited response to mecasermin treatment, suggesting that further studies are needed.

Trofinetide, a synthetic analogue of IGF-1 originally developed as potential treatment for stroke (88) has been successfully repurposed for RTT. A recent phase III clinical trial (NCT04181723) has shown that trofinetide improves behavioral, communication, and physical RTT symptoms (89, 90) and trofinetide has gained FDA approval in 2023. Trofinetide is the first FDA-approved drug for the treatment of RTT.

### 3.8 Statins/Lovastatin

Statins, commonly used to lower cholesterol, have widely been explored in NDDs due to their ability to reduce neuroinflammation and enhance synaptic function and neuronal survival [reviewed in (91)]. Several studies have suggested that cholesterol metabolism is perturbed in mice and individuals with RTT [reviewed in (58)]. Interestingly, initial research showed promising effects of statin treatment (fluvastatin and lovastatin) in RTT mice (92), but subsequent studies in a mouse model with a different genetic background yielded in conflicting results (93), illustrating the complexity of genetic and pharmacological interactions in NDDs like RTT. Therefore, personalized DR approaches may be necessary to optimize therapeutic outcomes in individuals with RTT. In 2015, a phase II open label dose escalating study of lovastatin in a small group of RTT patients (NCT02563860) has shown some preliminary positive results in improving visual recognition, memory and eye tracking.

### 3.9 Glucocorticoids/Corticosterone

Glucocorticoids are steroid hormones well known for their use in the treatment of inflammation, autoimmune diseases, and cancer. Corticosteroids (predominantly prednisolone and hydrocortisone) and adrenocorticotropic hormone (ACTH) are widely used as anti-epileptic drugs in pediatric populations, including RTT and CDD (94, 95). Interestingly, different studies have shown that pharmacological intervention with the glucocorticoid system using low-dose corticosterone has an impact on the symptoms and lifespan in an RTT mice (96, 97). Interesting pre-clinical studies have recently investigated the effects of pregnenolone-methyl-ether, a synthetic neuroactive steroid and derivative of pregnenolone on CDD cell and mouse models (98, 99). However, further research is needed to confirm these findings and determine the safety and efficacy of glucocorticoid therapies in humans with RTT and RTT-like disorders.

## 4 Discussion

RTT and RTT-like syndromes pose significant challenges for therapeutic strategies due to their rarity, complex etiology, and multifaceted symptomatology (Table 1). Traditional drug discovery pipelines often struggle to navigate these complexities. However, given the urgent need for effective treatments in RTT and related disorders, DR emerges as a pragmatic and resource-efficient strategy to expedite clinical translation and enhance treatment availability for affected individuals. In this discussion, we briefly examine the challenges and limitations of DR in RTT and RTT-related disorders and suggest potential solutions to advance the field.

One of the primary hurdles in drug development for RTT and RTT-like disorders undoubtedly lies in their rarity. Traditional drug development necessitates substantial financial investment to design, conduct, and analyze large-scale clinical trials involving significant numbers of participants.

Nevertheless, owing the rarity of RTT and related disorders, recruiting an adequate number of eligible participants for such trials can be extremely challenging and costly.

The heterogeneity and the diverse array of symptoms manifested by these disorders pose additional challenges. While the underlying genetic and molecular mechanisms overlaps to some extent, individual variability demands tailored therapeutic approaches. Grouping patients by shared clinical symptoms instead of genetic diagnosis, and developing DR strategies targeting symptoms that can likely be treated by DR candidates, such as proposed in the recently EU-funded SIMPATHIC project,^1^ may increase the efficiency of DR.

Repurposing existing drugs with pleiotropic effects or multitargeted mechanisms of action can address multiple aspects of the disorders simultaneously, offering holistic treatment approaches. Furthermore, combinatory repurposing targeting different pathways can be tailored to specific symptoms/domains which may lead to greater therapeutic benefits than any single agent alone. However, it is essential to note that combinatory therapies also pose challenges in terms of optimizing drug combinations, dosing regimens, and potential interactions. Rigorous preclinical and clinical studies are imperative to evaluate their safety and efficacy in RTT and RTT-like syndromes, considering the unique characteristics and variability of these disorders among affected individuals.

Rigorous preclinical and clinical studies are also crucial for better understanding the complex pathophysiology of these syndromes. To date, the precise molecular mechanisms underlying these complex disorders are still not fully understood; hindering the identification and validation of potential drug targets. This specifically applies to CDD and FOXG1-syndrome: both conditions were identified as distinct clinical entities only recently and it is understandable that research efforts initially focused primarily on “classical” RTT. This discrepancy is reflected also in the very different numbers of repurposing studies highlighted in Figure 1. Continued efforts in pre-clinical (identification of valuable cell and animal models etc.) and clinical research (better understanding of the natural history, clinical manifestations, disease progression, biomarkers etc.) will be essential for advancing our understanding and improving outcomes for individuals affected by these syndromes. In particular, better characterizing the shared symptoms and pathways across these entities, will provide valuable insights into the underlying biology and potentially uncover new common mechanisms and targeted therapies. If the disorders demonstrate convergence in their underlying molecular pathways, this provides an opportunity for designing joint DR strategies across RTT and RTT-like disorders. This could reduce the time needed for the development of DR and increase the number of patients benefiting from the treatments, resulting in more attractive business models.

Despite promising DR results in preclinical or early-phase clinical trials for RTT and related disorders in our opinion DR is still underrated and underutilized in this kind of disorders. DR holds immense potential for addressing the unmet medical needs and therapeutic challenges posed by such complex NDDs, and recent advancements screening and computational techniques, offer the unique opportunity to predict drug-disease interactions and prioritize candidate compounds for further investigation. By leveraging existing drugs and repurposing them for new indications, this approach offers a pragmatic and efficient strategy to accelerate the development of treatments for individuals affected by these debilitating conditions.

## Data availability statement

The original contributions presented in the study are included in the article/supplementary material, further inquiries can be directed to the corresponding author.

## Author contributions

CF: Writing−review and editing, Writing−original draft, Supervision, Project administration, Conceptualization. P’tH: Writing−review and editing, Writing−original draft. AM: Writing−review and editing, Writing−original draft. FE: Writing−review and editing, Writing−original draft. CV: Writing−review and editing, Writing−original draft.

## Footnotes

1
www.simpathic.eu
